# Profiling Microglia through Single-Cell RNA Sequencing over the Course of Development, Aging, and Disease

**DOI:** 10.3390/cells11152383

**Published:** 2022-08-02

**Authors:** Spyros Pettas, Korina Karagianni, Eirini Kanata, Athanasia Chatziefstathiou, Nikoletta Christoudia, Konstantinos Xanthopoulos, Theodoros Sklaviadis, Dimitra Dafou

**Affiliations:** 1 Department of Genetics, Development, and Molecular Biology, School of Biology, Aristotle University of Thessaloniki, 541 24 Thessaloniki, Greece; spyrospg@bio.auth.gr (S.P.); korinagk@bio.auth.gr (K.K.); chatziea@bio.auth.gr (A.C.); cnikolett@bio.auth.gr (N.C.); 2 Neurodegenerative Diseases Research Group, Department of Pharmacy, School of Health Sciences, Aristotle University of Thessaloniki, 541 24 Thessaloniki, Greece; ekanata@bio.auth.gr (E.K.); xantho@pharm.auth.gr (K.X.); sklaviad@pharm.auth.gr (T.S.)

**Keywords:** microglia, single-cell RNA sequencing, neurodegenerative diseases, aging, development, disease, microglial heterogeneity

## Abstract

Microglia are macrophages present in the brain that function as the primary and most important source of immune response in the central nervous system (CNS). Regardless of their multitasking role, our knowledge regarding their molecular heterogeneity is limited; due to technical restrictions, it is only possible to measure gene expression in cell populations, not individual cells, with the results reflecting average mRNA levels. Therefore, recent scientific approaches have focused on single-cell techniques such as single-cell RNA sequencing (scRNAseq), a powerful technique that enables the delineation of transcriptomic cell-to-cell differences, revealing subpopulations with distinct molecular and functional characteristics. Here, we summarize recent studies that focused on transcriptomic microglial subpopulation clustering and classify them into three distinct groups based on age, spatial distribution, and disease. Additionally, we cross-compare populations from different studies to identify expressional and functional overlaps between them.

## 1. Introduction

Microglia are the resident macrophages of the brain, acting as the primary and main source of immune response in the central nervous system (CNS). They enter the developing CNS at early embryonic stages [1,2] and proliferate until reaching 0.5–16.6% of the total cell numbers in the adult human brain [3] and 5–12% in the mouse brain [4]. Microglia display a multifunctional role in neuronal activity modulation in both homeostatic and pathophysiological conditions [5], using signaling mechanisms which are not yet fully described. During development, they affect several neuronal structures [6], promote synapse formation [7], and modulate neurogenesis by synapse pruning [8,9].

**Figure 1:**
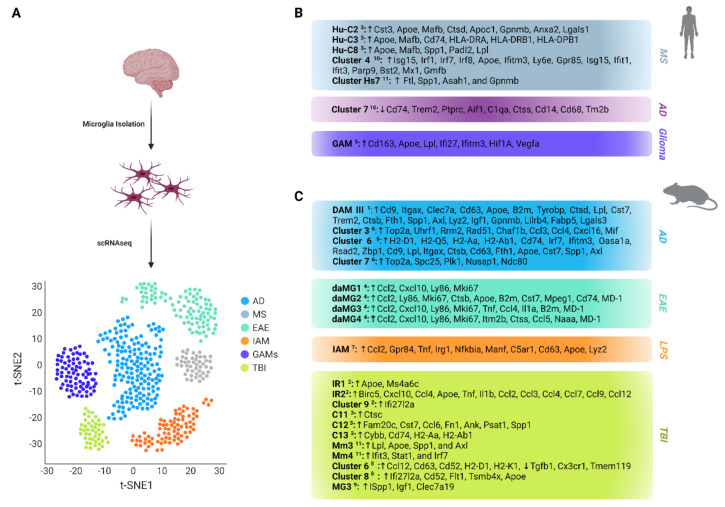
Schematic overview of the most distinct disease-associated microglial populations and their transcriptomic signatures revealed through transcriptional profiling of murine and human microglia using single-cell RNA sequencing. (**A**) Schematic representation of research process for molecular classification of microglial subpopulations. (**B**) Most regulated human disease-associated microglial populations and their transcriptomic signatures. (**C**) Most regulated mouse disease-associated microglial populations and their transcriptomic signatures. Microglial subsets are represented with superscript numbers as described in the studies of ^1^ Keren-Shaul et al. [14], ^2^ Hammond et al. [15], ^3^ Masuda et al. [16], ^4^ Jordão et al. [17], ^5^ Sankowski et al. [18], ^6^ Mathys et al. [19], ^7^ Sousa et al. [20], ^8^ Wicher et al. [21], ^9^ Li et al. [22], ^10^ Olah et al. [23], and ^11^ Miedema et al. [24]. AD: Alzheimer’s Disease, MS: Multiple Sclerosis, EAE: Experimental Autoimmune Encephalomyelitis, LPS: Lipopolysaccharide, IAM: Inflammatory-Associated Microglia, GAM: Glioma-Associated Microglia HIV: Human Immunodeficiency Virus, TBI: Traumatic Brain Injury. ↑ denotes transcriptomic upregulation, ↓ denotes transcriptomic downregulation. Figure created with BioRender.com (accessed on 22 June 2022).

Microglial activation is commonly considered to be any morphological or biochemical modification from the naive state, although polarization can be complex or tailored to a particular disease or developmental state. Despite their multitasking role, our knowledge about microglial molecular heterogeneity is limited due to technical restrictions, meaning that gene expression must be measured in cell populations instead of individual cells, reflecting average mRNA levels [10]. Therefore, recent scientific approaches have focused on single-cell profiling, such as single-cell RNA sequencing (scRNAseq), and its alternative technique, single-nucleus RNA sequencing (snRNAseq), revealing subpopulations with distinct molecular and functional characteristics (Figure 1A). scRNAseq is a powerful technique that enables the delineation of transcriptomic cell-to-cell differences [11]. It proven a useful tool for unbiased characterization in different cellular conditions through the identification of potential markers and transcriptional factors [12]. On the other hand, snRNAseq provides the ability to examine the transcriptional profile of frozen tissues, although its sensitivity is modest and it is unsuitable for identifying cellular activation in microglia [13].

The development of sequencing technologies has led to a variety of scRNAseq techniques being presented in recent years, greatly aiding our understanding of dynamic gene expression at the single-cell level. CEL-seq2, Drop-seq, MARS-seq, SCRB-seq, Smart-seq, and Smart-seq2 are six popular scRNAseq techniques [25]. Although a number of scRNAseq protocols and methods have been developed and each possess unique characteristics with distinct advantages and disadvantages, the typical steps for analysis are as follows: (a) single-cell isolation and lysis; (b) reverse transcription into cDNA; (c) amplification of cDNA; (d) generation of high-throughput sequencing libraries; (e) computational data processing (e.g., quality control, read mapping and expression quantification, normalization, imputation); and (f) bioinformatics analysis (e.g., subpopulation identification through unique molecular identifiers, differential expression, cell trajectory inference, network reconstruction) [26]. A significant step in bioinformatics analysis of high-dimensional single-cell data is the visualization of cell heterogeneity through clustering in two dimensions (Figure 1A) using nonlinear dimensionality reduction techniques such as t-distributed stochastic neighbor embedding (t-SNE) [27] and uniform manifold approximation and projection (UMAP) [28].

In this review, we summarize recent relevant studies, concentrating on microglial subpopulation clustering and further categorizing them into three distinct groups associated with age, region, and disease. Additionally, we cross-compare populations from different studies to identify expressional and functional overlaps between them.

## 2. Microglia Phenotypes in Homeostasis

Nowadays, the selection of microglial-relevant markers is a scientific difficulty because there is an enormous amount of information on numerous sets of markers that is available for such investigations. Thus, it is crucial to examine the functions and tissue state of certain microglial phenotypes with precision. Regardless of the present cell phenotype, certain general microglia markers can be found. The most frequently used homeostatic markers include the fractalkine receptor (*CX3CR1*), cluster of differentiation receptors (*CD68*, *CD11b*, *CD14*, *CD45*, *CD80*, and *CD115*), and ionized calcium-binding adapter molecule 1 (*IBA-1*). According to current knowledge, transmembrane protein 119 (*TMEM119*), purinergic receptor *P2Y12R*, olfactomedin-like 3 (*OLFML3*), and spalt-like transcription factor 1 (*SALL1*) are the most precise general homeostatic microglia core indicators [29].

## 3. Microglia Phenotypes in Diseases

Microglia resemble the macrophages of the brain and play a critical role in both homeostatic and neurodegenerative conditions. Due to their multifunctional role in the CNS, recent studies have focused on investigating their heterogeneity and identifying phenotypically and molecularly distinct subsets. These studies have revealed various disease-associated microglial populations with high plasticity and molecular heterogeneity, suggesting differences in their involvement in neurodegeneration (Figure 1B,C).

### 3.1. Microglia in Multiple Sclerosis

Multiple sclerosis (MS) is an auto-immune disease illustrated by inflammation and focal areas of demyelination, leading to axonal degeneration and neuronal death [24]. To test the inflammation in different stages of the disease, Miedema et al. performed scRNAseq in brain macrophages to study how these cells are affected in Normal Appearing White Matter (NAWM), considered to be the pre-clinical stage of the disease, and White Matter Lesions (WML), considered to be the clinical stage of the disease [24]. The results showed widespread microglial heterogeneity in the human MS brain. In addition to homeostatic microglia, the authors detected signs that they observed almost exclusively in NAWM or WML, showing disease-related signatures that overlap with those found in development and neurodegenerative diseases. Expression of myelinogenic, developmental, and disease-related microglial genes (*FTL*, *SPP1*, *ASAH1*, and *GPNMB*) indicative of an activated/phagocytic microglial phenotype was enriched in one of the clusters (cluster Hs7). These data indicate that cells in cluster Hs7 (Figure 1B) are microglia associated with demyelination or other myelin-related processes [24]. The expression of microglia marker genes (*CX3CR1, P2RY12, IRF8*) was depleted in this cluster and in fact, Hs7 microglia were specifically detected in lesion types with active demyelination.

To test the hypothesis that Hs7 cells are associated with demyelination, they profiled brain macrophages in a demyelinated and remyelinated cuprizone mouse model. Two clusters associated with demyelination were identified: Mm3 and Mm4 (Figure 1C). Cluster Mm3 expressed genes related to disease and development (*Lpl, Apoe, Spp1*, and *Axl*), and cluster Mm4 expressed genes associated with interferon responses (*Ifit3*, *Stat1*, and *Irf7*). Expression of the Mm3 marker gene set was significantly enriched in cluster Hs7, and in fact, both showed activation/phagocytosis profiles [24]. Taken together, this study points out that the activated/phagocytic microglia in WML is an early but constant disease response, associated with demyelination during lesion formation.

In the same direction, Masuda et al. isolated microglia from both healthy and disease mouse models in order to investigate their involvement in neurodegenerative and neuroinflammatory disorders. Consequently, microglial plasticity and response were assessed utilizing two different neurodegenerative mouse models: a model of toxic demyelination after cuprizone treatment and a facial nerve axotomy (FNX) disease model. The toxic demyelination model causes the loss of oligodendrocytes in the corpus callosum [30], while the FNX model leads to remote neurodegeneration within the facial nucleus [31]. Analysis revealed one cluster for neurodegeneration and two clusters for demyelination and remyelination conditions. scRNAseq in microglia from both neurodegeneration disease mouse models revealed three differentiated subpopulations, represented by clusters C11, C12, and C13 (Figure 1C). Cluster C11 was correlated with the FNX mouse model and high expression of *Ctsc* three days after FNX, while 14 days after FNX microglia recovered to homeostatic conditions. In contrast, C12 (demyelination) and C13 (remyelination) gene expression patterns remained almost unaltered after ten weeks of cuprizone treatment. C12 and C13 disease-associated clusters presented a range of expression markers in common, such as *Apoe*, *Axl*, *Igf1*, *Lyz2*, *Itgax*, *Gpnmb*, and *Apoc1*. C12 overexpressed *Fam20c*, *Cst7*, *Ccl6*, *Fn1*, *Ank*, *Psat1*, and *Spp1* and C13 showed an induced expression of *Cybb* and the MHC class II genes *Cd74*, *H2-Aa*, and *H2-Ab1*.

Further scRNAseq studies by Masuda and colleagues focused on human microglia isolated from healthy brain tissue, as well as tissue from histologically confirmed early MS patients. Clustering revealed four distinct classes of healthy microglia (HHu-C1 to HHu-C4) and seven classes from MS-related tissues (Hu-C2 to Hu-C8). Healthy HHu-C1 and HHu-C2 clusters showed strong upregulation of *CST3*, similarly to mouse C9 and C10 clusters, and the HHu-C4 cluster revealed high expression levels of the chemokine genes *CCL4* and *CCL2* and the zinc finger transcription factors *EGR2* and *EGR3*.

Early MS-related Hu-C5, Hu-C6, and Hu-C7 clusters consisted entirely of transcriptionally “healthy” microglial cells. These clusters showed the highest expression levels of the microglial core genes and were therefore considered to represent homeostatic states of microglia in early MS stages. The Hu-C4 cluster, which contained both homeostatic and MS-related microglia, was characterized by reduced expression levels of the core signature genes and elevated levels of *CCL2*, *CCL4*, *EGR2* and other chemokine and cytokine genes, which suggests that these microglia were pre-activated. Three clusters that were enriched in MS microglia (Hu-C2, Hu-C3, and Hu-C8) were separated from the homeostatic clouds on t-SNE plots [16]. These clusters showed an increased expression level of *APOE* and *MAFB*, whereas the expression of microglial core genes was downregulated or even absent (Figure 1B). The Hu-C2 cluster was characterized by high expression levels of *CTSD*, *APOC1*, *GPNMB*, *ANXA2*, and *LGALS1*. Hu-C3 microglia showed increased gene expression of MHC class II-related molecules, such as *CD74*, *HLA-DRA*, *HLA-DRB1*, and *HLA-DPB1*. This suggests an immunoregulatory role, reminiscent of the microglial subtype associated with remyelination in mice (C13). Finally, Hu-C8 showed strong expression of the *SPP1*, *PADI2* and *LPL*, genes, similar to C12 microglia associated with demyelination in mice. 

Of note, canonical correlation analysis of mouse and human microglia orthologues confirmed that clusters Hu-C2, Hu-C3, and Hu-C8 are associated with MS patients and have an expression profile similar to demyelination (C12) and remyelination (C13) clusters in FNX and cuprizone mouse models [16].

Emphasizing myeloid diversity during neuroinflammation, Jordão et al. performed scRNAseq in brain tissues from healthy and EAE (Experimental Autoimmune Encephalomyelitis) mice, one of the most common MS animal models [32]. Molecularly distinct classes and subclasses of microglial cells from perivascular space and parenchyma were identified. Clustering showed that microglia separated into two opposing states: homeostatic microglial (hMG) subsets in healthy mice and disease-associated microglial (daMG) subsets in EAE mice. Two populations, hMG1 and hMG2, were present under homeostatic conditions, whereas four subsets (daMG1, daMG2, daMG3, and daMG4) were present during disease (Figure 1C).

The results showed that all microglial populations (healthy and disease-associated) expressed *Bhlhe41*, *Gpr34*, *Sall1*, *Hexb*, *Olfml3*, *Siglech*, *P2ry12*, *P2ry13*, *Serpine2*, and *Sparc*. daMG subsets displayed lower expression of *P2ry12*, *Slc2a5*, and *Maf* and higher expression of *Ly86*, *Ccl2*, *Cxcl10*, and *Mki67*, showing proliferative potency [17]. Variations were detected in the expression of specific chemokines, cytokines, and cysteine proteases. The most inflammatory daMG subsets (daMG 2–4) strongly downregulated several core signature markers, such as *P2ry12*, *Tmem119*, and *Selplg*, and upregulated *MD-1*. *Olfml3* and *Sparc* were the only core microglial genes that remained stable through neuroinflammation, showing that these genes may serve as robust microglial markers in health and disease [17]. daMG2 presented high expression levels of *Apoe*, *Cd74*, and *Ctsb* and a low proliferation rate. On the other hand, daMG3 upregulated *Cxcl10*, *Tnf*, *Ccl4*, and APC genes, whereas daMG4 expressed high levels of *Ccl5*, *Ctss*, and *Itm2b*. daMG3 and daMG4 displayed high proliferative capacity. Upregulation of *Cd74* in daMG2, *Cxcl10* in daMG3, and *Ccl5* in daMG4 suggest an interaction between them, possibly at different activation stages [17].

### 3.2. Microglia in Alzheimer’s Disease

Alzheimer’s Disease (AD) is an age-related neurodegenerative disease characterized by progressive memory loss and cognitive dysfunction, which is usually histologically demonstrated by the accumulation of amyloid-beta (Aβ) plaques in the parenchyma, the formation of neurofibrillary tangles, and neuroinflammation [33]. 

Using a mouse model (5XFAD) that expresses five human familial Alzheimer’s disease (AD) gene mutations at different age stages, Keren-Shaul et al. identified two novel microglial subtypes, exhibiting a unique molecular expression signature related to AD and other neurodegenerative conditions, termed as disease-associated microglia (DAM). 

Massively parallel scRNAseq from both AD and wild type brain tissue revealed a map of ten distinct populations based on cluster-specific expression patterns of the 500 most variable genes. Among those populations, a large group of microglia were identified and further subcategorized into three subpopulations (I, II, and III). Microglia from groups II and III constitute distinctive microglial subsets associated only with the AD phenotype, in contrast with group I, which represents homeostatic microglia.

Further studies revealed significant differences in gene expression levels between homeostatic and DAMs, such as reduction of the purinergic receptors *P2ry12/P2ry13*, *Cx3cr1*, and *Tmem119* and upregulation of various known AD risk factors (*Apoe*, *Lpl*, *Tyrobp*, and *Trem2*) in DAM clusters. Additionally, DAMs express unique genes, such as *Cd9*, *Itgax* (*Cd11c*), *Clec7a*, and *Cd63*, indicating them as potential disease markers (Figure 1C). Gene set enrichment analysis (GSEA) showed a strong correlation of DAM-specific genes with lysosomal/phagocytic pathways, endocytosis, and regulation of the immune response [14]. Furthermore, single-cell sorting experiments reveal that DAM cells are detected specifically within the cortex and in proximity to Aβ plaques, highlighting their phagocytic and possible neuroprotective role in AD or other neurodegenerative diseases [14].

Through k-nearest neighbours (kNN) analysis from all major disease stages (1, 3, 6, and 8 months), this study pointed out that microglia can proceed from the homeostatic to the disease-associated phenotype through a transitional state as a function of disease progression [14]. Comparison between DAM clusters showed that cluster II appears as an intermediate disease subpopulation, expressing only a partial set of DAM-associated genes, including *Apoe*, *B2m*, *Tyrobp*, and *Ctsd*, through a TREM2-independent program [14]. On the other hand, cluster III expressed all DAM-associated genes in a TREM2-dependent program, including lipid metabolism and phagocytic pathway genes such as *Lpl*, *Cst7*, and immunoreceptor *Trem2*.

In the same framework, Mathys et al. determined the transcriptomic diversity of microglial cells isolated from the hippocampus of CK control and CK-p25 AD-like mice, at four timepoints (0, 1, 2, and 6 weeks after p25 induction) during the progression of neurodegeneration. Clustering revealed multiple distinct microglial subsets (Clusters 1–8) constituted of cell populations from specific time points. Thus, cluster 2 contained cells isolated from the CK control and 0-week CK-p25 mice, clusters 3 and 7 mainly included 1-week CK-p25 cells, and cluster 6 was composed of 2-week and 6-week CK-p25 cells. This clustering exhibited various microglial cell states during neurodegeneration that grouped separately from microglia isolated from CK control mice. Therefore, these neurodegeneration states were divided into two groups: an early-response state formed mainly from 1-week CK-p25 cells (Clusters 3 and 7) and a late-response state composed mainly of 2-week and 6-week CK-p25 cells (Cluster 6).

A comparison of early response (cluster 3 and 7) and late response (cluster 6) states with homeostatic microglia (cluster 2) pointed out how microglia respond over time in neurodegeneration progress. Gene Ontology (GO) analysis showed that early response cluster 3 was characterized by over-represented cell-cycle and DNA replication and by repair genes such as *Top2a*, *Uhrf1*, *Rrm2*, *Rad51*, and *Chaf1b*. Cell cycle-related genes such as *Top2a*, *Spc25*, *Plk1*, *Nusap1*, and *Ndc80* were upregulated in cluster 7 as well. These clusters displayed significant differences in cell cycle phases, indicating high proliferation in response to neurodegeneration [19].

In contrast to clusters 3 and 7, cell cycle genes were not represented in late response cluster 6, in which immune response-related genes were over-expressed instead (Figure 1C). Genes that were upregulated include MHC I (*H2-D1*, *H2-Q5*) and MHC II (*H2-Aa*, *H2-Ab1*, *Cd74*) components, many interferon response genes, such as *Irf7* and *Ifitm3*, and genes related to the GO term “defense response to virus”, such as *Oasa1a*, *Rsad2*, and *Zbp1*. A comparison of early-response cluster 3 and late-response cluster 6 revealed that a large fraction of genes, including *Ccl3*, *Ccl4*, *Cxcl16*, and *Mif*, were significantly over-expressed in both clusters. 

As previously mentioned, cluster 6 was characterized by its antiviral and interferon response gene expression module, although there was a notable variation over individual cells, with a subset displaying a fold induction module score higher than the average. In a subset of cells, the MHC II module was at least one order of magnitude higher than the average, whereas in another subset ribosomal protein-encoding genes presented a much smaller distribution. Thus, these data suggest the existence of at least two different reactive microglial phenotypes in neurodegeneration [19].

Additionally, in a study conducted by Olah et al., a unique subset of microglia (microglial cluster 7) has been detected that is characterized by the enrichment of genes usually lacking in AD patients (Figure 1B). In detail, this study investigated the microglial population purified from human cerebral cortex samples obtained at autopsy and during neurosurgical procedures. Using scRNAseq from 16,242 cells, they identified nine human microglial subpopulations based on their expression of microglia-enriched markers (nine microglial clusters, named cluster 1–9) such as *C1QA*, *C1QB*, *C1QC*, and *GPR34*. The results revealed that clusters 1 and 2 represent homeostatic microglial states, implementing housekeeping tasks of the CNS parenchyma. Cluster 3 appears as a subset enriched in genes related to cellular stress. Cluster 4 is enriched in genes related to the interferon response signaling pathway (*IRF1*, *IRF7* and *IRF8*), while clusters 5 and 6 overexpress genes such as *IL-10*, *IL-4*, and *IL-13* that are related to anti-inflammatory responses. Cluster 8 exhibits the highest expressional diversity of transcriptional factors and cell surface molecule encoding genes, whereas cluster 9 is enriched in genes associated with the cell cycle (*CREB* and *ATF*), suggesting that it may constitute a pool of proliferating microglial cells [23]. Of note, the cluster 7 transcriptome profile showed high upregulation of genes related to antigen presentation, such as *CD74*, and displayed a frequency reduction in AD tissues.

### 3.3. Microglia in Inflammation

As microglia sense and disseminate inflammatory signals, they coordinate immune responses [34]. Although neuroinflammation is frequently connected to neurodegeneration, the inflammatory response itself serves as a primary, temporary and self-limiting defense mechanism [34]. 

In order to profile microglia under inflammatory conditions, Sousa et al. performed scRNAseq in the CNS of LPS-induced mice to mimic inflammatory and infectious conditions, with saline-injected mice used as controls. Clustering revealed three distinct microglial subpopulations, one related to saline injection (control) and two related to LPS injection (“main LPS” and “subset LPS”), named inflammatory-associated microglia (IAMs). GO term analysis of upregulated genes in LPS-injected microglia showed “translation”, “protein folding”, “ribosome biogenesis”, and “immune system process” involvement, whereas downregulated genes uncovered a significant enrichment in “regulation of TGF-β receptor signaling pathway” [20]. IAMs displayed significantly lower expression of microglial homeostatic genes, such as *P2ry13/P2ry12*, *Tmem119*, *Mef2c*, *Fcrls*, *Gpr34*, and *Singlech*, whereas classical pro-inflammatory genes such as *Ccl2*, *Gpr84*, *Tnf*, *Irg1*, and *Nfkbia* were upregulated (Figure 1C). The “Subset LPS” cluster was identified closer to the naïve microglial cluster, suggesting that they represent either a group of microglia that are less sensitive to inflammatory stimuli or a group that has already recovered [20]. “Subset LPS” and “main LPS” clusters showed different expression patterns compared to the control cluster. A comparison of the two IAM clusters showed that the “main LPS” cluster was characterized by higher expression of *Manf* and *C5ar1*, whereas the “subset LPS” cluster upregulated *Stab1* and *Ash1l*. Overall, IAMs show a unique differentially expressed gene pattern, highlighting the microglial heterogeneity of activation states under inflammatory conditions [20].

### 3.4. Microglia in Traumatic Brain Injury

Traumatic brain injury (TBI) represents one of the most substantial causes of fatality and disability in ages under 40 years in developed countries [35,36]. It is known that brain injury can trigger inflammatory responses, leading to neurodegeneration and other long-term impairments. New evidence suggests that microglial activation in response to injury may have reparative/restorative effects. Several studies have investigated the impact of microglia in brain injury models in order to delineate their role and their utilization in a therapeutic approach [37].

Hammond et al. analyzed a great number of white matter isolated microglial cells after TBI, derived from mice exposed to a focal demyelinating injury caused by LPC injection. Saline-injected adult (P100) mice samples and untreated P100 whole-brain samples were used as control states. Two main distinct injury-responsive microglial subtypes were revealed, IR1 and IR2 (Figure 1C). The IR1 cluster mostly consisted of whole-brain and saline-injected control microglia, representing homeostatic microglial conditions. Conversely, the IR2 cluster was exclusively composed of microglia from LPC-injected demyelinated lesions, displaying the activation microglial program after injury. In more detail, IR2 showed remarkable downregulation of the canonical microglial markers *P2ry12* and *Cx3cr1* and distinctive over-expression of *Birc5* (a cell proliferation marker), *Cxcl10* (an interferon response gene), *Ccl4*, and *Apoe* [15].

To better understand microglial activity in damage, Witcher et al. contacted scRNAseq on mouse brains after TBI at a critical time point (7dpi) in the development from acute to chronic pathogenesis. Increased inflammation and type-1 interferon signaling was linked to TBI’s influence on microglia. A subset of microglia (Cluster 6) expressed significant levels of interferon-responsive genes (*Ifitm3*, *Isg15*, *Ifi27l2a*) and other immune-related genes (*Ccl12*, *Cd63*, *Cd52*, *H2-D1*, and *H2-K1*), with a corresponding reduction in homeostatic genes (*Tgfb1*, *Cx3cr1*, and *Tmem119*), whereas cluster 8 was characterized by interferon- (*Ifi27l2a*) and damage-related genes (*Cd52*, *Flt1*, *Tsmb4x*, *Apoe*) (Figure 1C). According to these findings, microglia are key mediators of chronic inflammation and restrict neuronal homeostasis at the transcriptional, structural, physiological, and functional levels [21].

Further scRNAseq and functional studies contacted by Li et al. uncovered a unique microglial subset (injury-induced neonatal MG3 microglia), found only in neonatal mice, that orchestrates the injury response process and can lead to scar-free healing of spinal cord injuries. In more detail, when activated in response to injury, MG3 microglia can temporarily secrete fibronectin, a protein that forms scaffolds of extracellular matrix in order to ligate the injured ends of the spinal cord and express peptidase inhibitors involved in the termination of the inflammatory process [22]. MG3 microglia express several genes commonly found in disease-associated, proliferative region-associated (*Spp1*, *Igf1*, and *Clec7a19*), and embryonic microglial cells (*Ms4a7*, *Ms4a6c*, and *Lgals1*), suggesting that a subpopulation of de-differentiated homeostatic microglia are being activated during injury in order to repair the wound and restore homeostasis [22]. 

### 3.5. Microglia in Glioma

Gliomas are diffusely developing brain tumours that arise from astroglial or oligodendroglial progenitor cells, and are characterized by strong innate immune infiltration of various myeloid cells [18]. To profile human microglia under this condition, Sankowski et al. used non-diseased access tissue from patients undergoing brain surgery to remove malignant glioma. 

scRNAseq revealed nine major subpopulations (Clusters C1-C9) with variations over the transcriptional spectrum. C3 upregulated the microglial core genes (e.g., *CX3CR1* and *TMEM119*), C2 strongly expressed MHC-II and antiviral immunity genes (e.g., *HLA-DRA*, *CD74* and *IFI44L*), and C6 and C7 downregulated *CX3CR1* and upregulated integrin-receptor-binding protein and metabolism genes (e.g., *SPP1*, *APOE* and *IL1B*) [18]. GO term analysis showed that clusters C2, C6, and C7 expressed MHC-II-associated genes corresponding to “antigen processing and presentation of peptide antigen”. Clusters C1, C5, C8, and C9 revealed GO term enrichment for “positive regulation of MAPK cascade” and “positive regulation of chemotaxis” [18].

Furthermore, the same study revealed the existence of disease-associated microglia clusters in human gliomas. Microglia were isolated from primary glioblastomas and compared with four age-matched controls. Fourteen distinguishable clusters (C1-C14) were created after the analysis. Clusters C9, C11, and C12 were control-enriched, clusters C3-C7, and C10 were mixed, and clusters C13 and C14 were almost exclusively composed of glioma-associated microglia (GAMs). C13 and C14 were characterized by lower expression of microglial core genes such as *CX3CR1* and *SELPLG* and higher expression of metabolic, inflammatory, and interferon-associated genes, such as *CD163*, *APOE*, *LPL*, *IFI27*, and *IFITM3* (Figure 1B). Mixed clusters C3–C7 and C10 showed similar expressions of *Spp1* compared with C13, higher than control-enriched C9, C11, and C12 clusters. GO term analysis revealed enrichment of the term “positive regulation of vasculature development” in clusters C10 and C14. The remaining GAM-associated clusters revealed enrichment of the GO term “antigen processing via MHC class I” [18].

## 4. Microglia Phenotypes in Development and Aging

**Figure 2:**
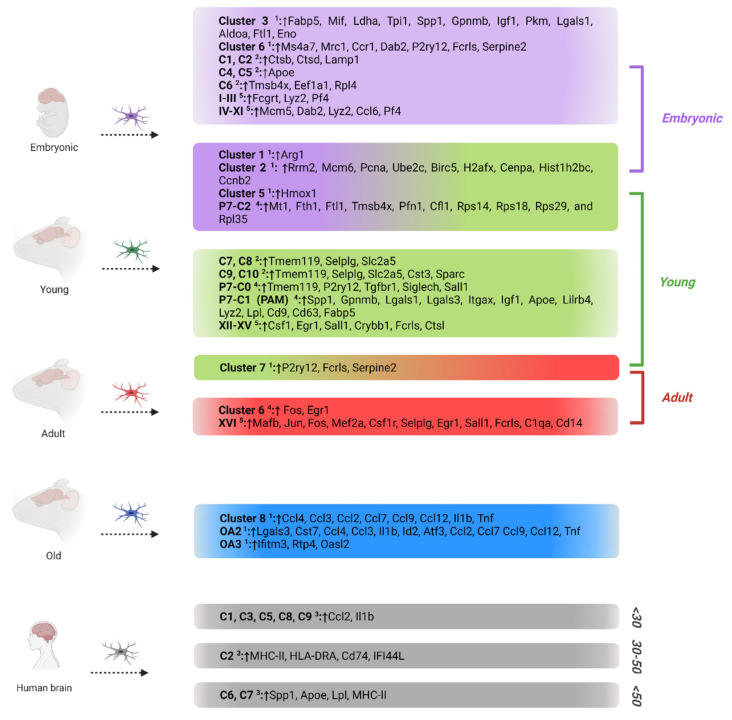
Overview of the most distinct age-associated microglial populations and their transcriptomic signatures revealed through transcriptional profiling of murine and human microglia using single-cell RNA sequencing. Microglial subsets are represented with superscript numbers as described in the studies of ^1^ Hammond et al. [15], ^2^ Masuda et al. [16], ^3^ Sankowski et al. [18], ^4^ Li et al. [39], ^5^ Matcovitch-Natan et al. [40]. ↑ denotes transcriptomic upregulation. Figure created with BioRender.com (accessed on 18 June 2022).

Although much of the study on microglia has focused on adult brain activities, data from developmental and aging research suggest that the nature of these processes changes over time. Aging, rather than inducing a universal program, drives a distinct transcriptional course in each cell population [38]. Thus, scRNAseq has revealed microglial heterogeneity during development and aging. Recent studies have exhibited gradual and diverse expression patterns among distinct microglial subtypes, highlighting different functions (Figure 2). 

Matcovitch-Natan et al. identified sixteen different microglial populations (I–XVI) across different temporal phases. Four main age-related microglial categories were formed: microglia derived from the yolk sac (I-III), embryonic microglia from different time points, termed early microglia (IV-XI), pre-microglia, which include all postnatal age stages (XII-XV), and adult microglia (XVI).

Most of the microglial subsets expressed a characteristic gene pattern, pointing out their temporal specificity and dynamics across different developmental stages, while several classes showed partial expression overlap (Figure 2). *Mcm5* was uniquely expressed in early microglial cells, *Csf1* displayed a pre-microglial marker, while *Mafb*, *Jun*, *Fos*, and *Mef2a* were strongly correlated with adult microglia [40]. Clusters I–III highly expressed *Fcgrt*, *Lyz2*, and *Pf4*, which were found to be expressed in several of the early microglial clusters (IV and V) as well. *Dab2* and other cell cycle regulatory and chromatin remodeling genes were found to be specifically expressed in early microglial classes, but not at later time points. Canonical transcriptional factors such as *Egr1* and *Sall1* were initially expressed in pre-microglia subpopulations and further induced in adulthood [40]. 

Within the same framework, Hammond et al. analyzed a great number of microglial cells isolated from mice during development (embryonic E14.5, early postnatal P4/5, late juvenile stage P30, adulthood stage P100, and aging P540). Clustering revealed nine differentiated microglial subtypes across all ages and conditions, termed clusters 1–9, and two clusters of old age microglia (OA2 and OA3 (Figure 2)). 

The embryonic and early postnatal stages (E14.5 and P5) showed the greatest diversity, whereas aging clusters showed a major redistribution of microglial states [15]. Homeostatic microglial genes such as *Fcrls*, *P2ry12*, *Cx3cr1*, *Trem2*, and *C1qa* were significantly expressed by most of the microglial clusters, while only *C1qa*, *Fcrls*, and *Trem2* were universally expressed. In contrast, clusters 3 and 4 showed an important downregulation of *P2ry12*, *Cx3cr1*, and *Tmem119* transcripts. Younger-aged clusters displayed various unique markers, such as *Arg1* (cluster 1), *Rrm2*, *Mcm6*, and *Pcna* (cluster 2a), *Ube2c*, *Birc5*, and *H2afx* (cluster 2b), *Cenpa*, *Hist1h2bc*, and *Ccnb2* (cluster 2c), *Fabp5* (cluster 3), *Spp1* (cluster 4), *Hmox1* (cluster 5), and *Ms4a7* (cluster 6). Cluster 3 overexpressed a wide range of other genes, such as *Mif*, *Ldha*, *Tpi1*, *Spp1*, *Gpnmb*, *Igf1*, *Pkm*, *Lgals1*, *Aldoa*, *Ftl1*, and *Eno1*, highlighting their association with glycolysis and potential functional overlap with macrophages in cell growth, motility, inflammation, and immunomodulation [41,42,43]. Cluster 4 displayed an expression pattern similar to cluster 3, suggesting a possible relationship and interaction. Remarkably, cluster 6 displayed a unique expression profile similar to brain border macrophages (*Mrc1*, *Ccr1*, *Dab2*) while at the same time over-expressing transcripts found in mature microglia (*P2ry12*, *Fcrls*, *Serpine2*). This suggests the idea that cluster 6 represents an intermediate state between microglia and their brain border neighbours, which comes to a symphony with the idea that these two cell types are derived from the same pool of yolk sac hematopoietic progenitors and migrate to the brain at the same time in development [44]. In addition, the aged microglia population was found predominantly in cluster 8, which highly expressed the chemokine *Ccl4*.

Two old age microglial clusters were identified (OA2 and OA3) in Hammond’s et al. study. OA2 microglia uniquely expressed several inflammatory signals that were absent in clusters 1–9. This cluster showed high expression of *Lgals3*, *Cst7*, *Ccl4*, *Ccl3*, *Il1b*, *Id2*, and *Atf3* (Figure 2). Cluster OA3 overexpressed interferon-response genes, including *Ifitm3*, *Rtp4*, and *Oasl2* (Figure 2). These genes can modulate inflammation [45], suggesting a potential inflammatory role in the aged brain. 

Likewise, by performing scRNAseq in six regions (cortex, striatum, cerebellum, olfactory bulb, hippocampus, and choroid plexus) of embryonic (E14.5), postnatal (P7), and adult (P60) brain tissues, Li et al. uncovered microglial heterogeneity among different developmental stages. Clustering revealed seven microglial populations (clusters 0–6) expressing microglial signature genes, such as *P2ry12* and *Salc2a5*. 

Within these clusters, adult microglia were mainly present in clusters 0 and 6. These clusters showed similar expression levels of homeostatic microglial signature genes, with *P2ry12* slightly lower in cluster 6. Cluster 6 was characterized by IEG expression, such as *Fos* and *Egr1* (Figure 2). Postnatal microglia were distributed among clusters 0–5, with dividing cells separated into two clusters depending on their cell cycle phases (G2/M phase microglia in cluster 3 and S phase microglia in cluster 4) [39]. Cluster 5 was enriched by embryonic cells, whereas the remaining microglia were grouped in clusters 1 and 2.

In scRNAseq analysis, cell cycle genes can hide cell-to-cell variations, masking functional relevant differences. Bearing this in mind, Li et al. re-clustered P7 microglial cells by removing cell cycle effects. The results revealed three clusters, P7-C0, P7-C1, and P7-C2, showing early postnatal microglial heterogeneity. P7-C2 was characterized by lower expression of microglial core genes (e.g., *Tmem119*, *P2ry12*, *Tgfbr1*, and *Selplg*) and higher expression of *Mt1*, *Fth1*, *Ftl1*, *Tmsb4x*, *Pfn1*, *Cfl1*, *Rps14*, *Rps18*, *Rps29*, and *Rpl35* (Figure 2). Microglia from clusters P7-C0 and P7-C1 expressed homeostatic genes (e.g., *Tmem119*, *P2ry12*, *Tgfbr1*, *Siglech*, and *Sall1*), with P7-C1 expressing them at lower levels. 

Along the same lines, Masuda et al. revealed distinct microglial populations across different developmental stages and brain regions. Clustering revealed thirteen distinct microglial subsets, with ten of them (C1–10) related to age and development. To investigate spatial and temporal microglia heterogeneity, Masuda and colleagues performed massively parallel single-cell analysis in microglial cells from multiple regions of the embryonic (16.5), juvenile (3 weeks), and adult (16 weeks) mouse CNS.

Examination of microglial transcriptional heterogeneity during development showed two major developmental stages with distinct expression patterns: embryonic microglia and postnatal microglia (including cells from both juvenile and adult mouse brains). Clusters C1-C6 mainly consisted of embryonic microglia and were distributed across the forebrain and cerebellum. C1 and C2 showed high expression of the lysosome-related genes *Ctsb*, *Ctsd*, and *Lamp1*, suggesting increased lysosomal activity [16] (Figure 2). Gene expression levels in the C1, C4, and C5 clusters revealed strong upregulation of *Apoe*, whereas in C6 the microglial subset genes *Tmsb4x*, *Eef1a1*, and *Rpl4* appear to be overexpressed [16] (Figure 2).

On the other hand, clusters C7-C10, which represent postnatal microglial populations, demonstrated altered transcriptional profiles compared to the embryonic microglial classes and variable distribution range across the cortex and cerebellum. Homeostatic genes such as *Tmem119*, *Selplg*, and *Slc2a5* were highly expressed across all postnatal microglial subpopulations. In addition, the C9 and C10 clusters showed high expression of *Cst3*, a gene that is associated with neurodegenerative diseases, and *Sparc*, which encodes a cysteine-rich acidic matrix-associated protein (Figure 2).

Sankowski’s et al. study revealed the influence of age on microglial phenotype. Patients were divided into three age groups: <30 years old, 30–50 years old, and >50 years old. Interestingly, clusters C1, C3, C5, C8, and C9 were enriched in <30 years old patients, clusters C6 and C7 were enriched in the >50 years old group, and cluster C2 was more involved in 30–50 years old patients [18] (Figure 2).

In summary, a variety of molecularly distinct microglial subsets were identified, suggesting different involvement in development and age progression. These studies underline the importance of better microglial classification in order to further understand their functions and mechanisms across different developmental stages.

## 5. Microglia Phenotypes in Brain Regions

Previous studies have demonstrated that microglia have distinct region-dependent transcriptional identities that vary regionally [46]. Region-dependent microglial heterogeneity was detected in Sankowski et al. study. They found two distinct anatomical locations in grey and white matter where microglia were molecularly different. White matter microglia showed higher expression of immune markers compared to grey matter microglia [18]. Expression of *HLA-DR* and *CD68* was significantly higher in white matter microglia, suggesting an involvement in oligodendrocyte maintenance [47]. Grey matter microglia were represented in MHC-II^low^ C3 and C8 clusters, whereas white matter microglia were overrepresented in MHC-II^high^ C2, C5, C6, and C7 clusters [18].

Another region-dependent microglial subset was characterized by Hammond et al. (2019). This microglial population was detected only in the early postnatal brain and was identified specifically in the subcortical axon tracks of the corpus callosum in the forebrain and cerebellum (ATM—Axon Tracks Microglia), regulating the growth and fasciculation of axons and refining synapses in a circuit- and region-specific manner. This cluster was further characterized by high enrichment of genes associated with immune cell activation, lysosomal activity, and phagocytosis, such as *Igf1*, *Gpnmb*, *Lgals1*, *Lgals3*, *Lamp1*, and *Cd68*, suggesting an involvement in phagocytosis of the brain materials in these specific regions [15].

Finally, Li et al. mentioned that microglial cells in the P7-C1 cluster mainly appeared in regions with cellular proliferation, such as developing white matter. This subset expressed high levels of *Igf1* and *Itgax* (Figure 2). Because of this, P7-C1 microglia were named proliferative region-associated microglia [39] (PAM).

## 6. Discussion

Overall, these studies display the high molecular heterogeneity of the microglial cell population in CNS. A combination of microglial single-cell analyses revealed more than 200 clusters, which we categorized into three main groups associated with age, region, and disease. Clusters within these groups share common microglial core signature gene expression, such as *P2ry12*, *P2ry13*, *Cx3cr1*, *Tmem119*, *Selplg*, and *Slc2a5*, at different levels in both humans and mice.

In addition to these core genes, each microglial subtype expresses different transcriptomic patterns related to its specific functional role in CNS, confirming multiple definable states of microglia over the course of age, spatial distribution, and disease. Interestingly, certain genes show a strong correlation with a variety of microglial clusters, belonging to the same main group and participating in similar molecular mechanisms. Genes such as *Cxcl10*, *Clec7a*, *Ctsb*, *B2m*, *Ccl6*, *Axl*, *Apoc1*, *H2-Aa*, and *H2-Ab1* have been found to be linked specifically with disease-associated microglia. Furthermore, young-aged microglia seem to have enriched expression of *Igf1*, *Ftl1*, *Dab2*, and *Lamp1*, whereas old-aged microglia seem to share similar gene expression with microglia detected in disease conditions (e.g., *Ccl4*, *Ccl3*, *Ccl7*, *Ccl9*, *Ccl12*, *Tnf*, *Ifitm3*, *Cst7*). This transcriptional pattern indicates that these genes may function as potential markers under different conditions, defining specific microglial states. 

**Figure 3:**
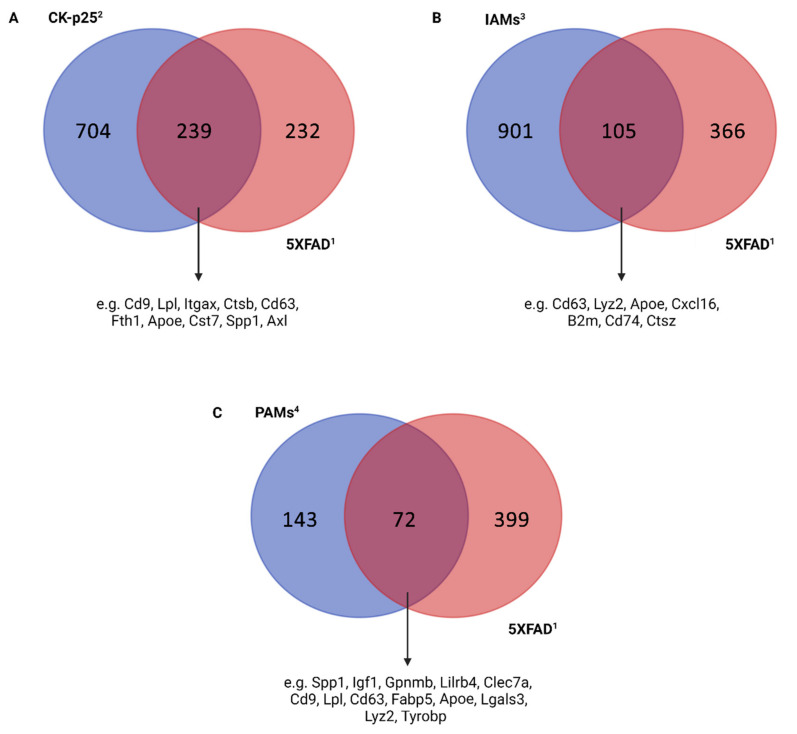
Comparison of upregulated genes between ^1^ Keren-Shaul et al. [14] DAM III cluster with different microglial subsets derived through single-cell RNA sequencing uncovers expressional and functional overlaps between them. Only upregulated genes with statistical significance (*p*-value < 0.05) were used for the comparison. (**A**) Venn diagrams depicting common and differential transcriptomic patterns between ^1^ Keren-Shaul et al. [14] DAM III cluster and ^2^ Mathys et al. [19] late-response cluster 6. (**B**) Venn diagrams depicting common and differential transcriptomic patterns between ^1^ Keren-Shaul et al. [14] DAM III cluster and ^3^ Sousa et al. [20] IAM cluster. (**C**) Venn diagrams depicting common and differential transcriptomic patterns between ^1^ Keren-Shaul et al. [14] DAM III cluster and ^4^ Li et al. [39] PAM clusters. Superscript numbers refer to the respective studies. Figure created with BioRender.com (accessed on 18 June 2022).

Across disease-associated microglia, we found a significant association between the DAM clusters of Keren’s et al. and Mathys’ et al. late-response cluster 6 (Figure 3A). These clusters shared common transcriptional signatures of upregulating genes, including *Cd9*, *Lpl*, *Itgax*, *Ctsb*, *Cd63*, *Fth1*, *Apoe*, *Cst7*, *Spp1*, and *Axl*. The expression profile between cluster 6 and DAM III was even more similar, indicating that these clusters represent an advanced stage of neurodegeneration. It is interesting to note that research over the past 20 years has revealed both positive and negative impacts of microglia in AD. The detection of DAM cells in proximity to Aβ plaques in the study by Keren et al. demonstrated their potential possible neuroprotective role in AD. However, microglia have been reported as detrimental in tau models of AD [48]. Taken together, it is unclear whether microglial function in neurodegenerative illnesses is beneficial but insufficient, or whether these cells are effective in the early stages of the disease and then lose their effectiveness or possibly become harmful later. In addition, Keren-Shaul et al. performed single-cell analysis in an ALS (Amyotrophic Lateral Sclerosis) mouse model, and the results showed that DAM cells were present in other neurodegenerative conditions as well, displaying almost the same conserved gene markers and involved in the same pathways as previously described.

Despite the fact that DAM clusters demonstrate various similarities with other disease-associated subtypes, distinct gene expression patterns may appear due to different pathological situations. For instance, DAMs and Sousa’s et al. IAM clusters both represent neuropathological conditions (Figure 3B). Although these clusters showed a few similarities in several genes (e.g., upregulation of *Cd63*, *Lyz2*, and *Apoe* and downregulation of *P2ry12*, *P2ry13*, and *Tmem119*), significant differences were noticed. For example, *Tyrobp* and *Trem2* were highly downregulated in IAMs and over-represented in DAMs. Based on expression profiling, IAMs showed elevated inflammatory reactivity, whereas DAMs displayed phagocytic and lysosomal gene identity. These distinct expression patterns may reflect different functional approaches to inflammation and neurodegeneration. Collectively, these results suggest that microglia under inflammation show molecular signals which are different from disease-associated profiles, supporting the idea that microglial modulation takes place through a very specific regulatory program [20].

Of note, high transcriptional overlap between DAMs and Li et al.’s PAMs was detected (Figure 3C). Both clusters upregulated *Spp1*, *Igf1*, *Gpnmb*, *Lilrb4*, *Clec7a*, *Cd9*, *Lpl*, *Cd63*, *Fabp5*, *Itgax*, *Lgals3*, *Apoe*, *Lyz2*, and *Tyrobp* and downregulated the microglial core genes *P2ry12* and *Tmem119*. Although these clusters derive from distinct functional categories, similarities between them support the idea that gene expression during development matches the transcriptional profile of aging and neurodegeneration through reactivation of certain genes [49].

Regarding age-associated microglial subtypes, studies have revealed higher heterogeneity between young-aged clusters compared to old-aged microglia [15,40]. This is in agreement with the fact that microglia in the early stages of development remain able to differentiate and demonstrate higher plasticity and adaptation [50]. On the other hand, microglial cells in advanced age seem to present a malfunctioning phenotype related to pathogenesis, perhaps due to the loss of this flexibility [50]. For instance, the old-aged clusters 8 and OA2 from Hammond et al. share a common gene expression profile with the injury-response cluster IR2 (e.g., *Ccl2*, *Ccl3*, *Ccl4*, *Ccl7*, *Ccl9*, *Ccl12*, and *Tnf*).

It is worth mentioning that protocols for enzymatic dissociation of brain tissue as well as different isolation approaches could possibly affect the gene expression signature. Microglial cells, which demonstrate elevated plasticity and dimensionality, are considered to be particularly sensitive to stimulation of their microenvironment, and thus are highly susceptible to ex vivo gene expression artifacts. As a result, it is critical to establish methods to prevent this aberrant transcriptional state. Recent studies have developed new digestion protocols using cold-active enzymes [15,51]. Notably, a protocol utilizing the addition of transcriptional and translational inhibitors during multiple steps of the dissociation process has been proposed as a more sufficient way to eliminate this problem [52].

## 7. Conclusions

In conclusion, scRNAseq techniques have shown for the first time the increased variation in microglial cell populations, especially in humans, where heterogeneity is significant, in contrast to mouse and other species models, which demonstrate one largely dominant microglial type [53]. Studies have revealed differences in gene expression levels under a variety of conditions. This molecular diversity can help us to better understand neuropathologies and design efficient therapeutic strategies to treat neurodegenerative and neuroinflammatory diseases based on the determined targeting of a microglial subset. In addition, a recent study of single-cell gene expression analysis in CSF of patients with relapsing-remitting MS (RRMS) and anti-myelin oligodendrocyte glycoprotein (MOG) disorder revealed that a CSF-specific microglia cell population may be involved in antigen presentation, with a similar gene expression profile to parenchymal microglia [54]. This shows that scRNAseq can serve as a powerful diagnostic technique for neurodegeneration by examining the CSF of patients.

Instances of inflammatory processes in the aging and diseased brains highlight the significance of proper microglial phenotypic description. The data presented in this study provide a thorough and useful overview of the state of the art and simplify the selection of more appropriate and precise markers for in-depth investigation of microglia and neuroinflammatory pathways in diverse pathophysiological circumstances. Recently, regulating microglial phenotypes has emerged as a treatment strategy in several CNS illnesses. The data acquired here are useful for selecting the right descriptors or study goals and will enable more accurate identification of microglial states in research while describing immunological pathways in the brain. In any case, our knowledge, for now, remains limited, and further study is needed.

## Data Availability

Data are available through the original studies.

## References

[1] Reemst K., Noctor S.C., Lucassen P.J., Hol E.M. (2016). The Indispensable Roles of Microglia and Astrocytes during Brain Development. Front. Hum. Neurosci..

[2] Ginhoux F., Greter M., Leboeuf M., Nandi S., See P., Gokhan S., Mehler M.F., Conway S.J., Ng L.G., Stanley E.R. (2010). Fate Mapping Analysis Reveals That Adult Microglia Derive from Primitive Macrophages. Science.

[3] Lawson L.J., Perry V.H., Gordon S. (1992). Turnover of Resident Microglia in the Normal Adult Mouse Brain. Neuroscience.

[4] Mittelbronn M., Dietz K., Schluesener H.J., Meyermann R. (2001). Local Distribution of Microglia in the Normal Adult Human Central Nervous System Differs by up to One Order of Magnitude. Acta Neuropathol..

[5] Bessis A., Béchade C., Bernard D., Roumier A. (2007). Microglial Control of Neuronal Death and Synaptic Properties. Glia.

[6] Marín-Teva J.L., Dusart I., Colin C., Gervais A., Van Rooijen N., Mallat M. (2004). Microglia Promote the Death of Developing Purkinje Cells. Neuron.

[7] Parkhurst C.N., Yang G., Ninan I., Savas J.N., Yates J.R., Lafaille J.J., Hempstead B.L., Littman D.R., Gan W.B. (2013). Microglia Promote Learning-Dependent Synapse Formation through Brain-Derived Neurotrophic Factor. Cell.

[8] Paolicelli R.C., Bolasco G., Pagani F., Maggi L., Scianni M., Panzanelli P., Giustetto M., Ferreira T.A., Guiducci E., Dumas L. (2011). Synaptic Pruning by Microglia Is Necessary for Normal Brain Development. Science.

[9] Schafer D.P., Lehrman E.K., Kautzman A.G., Koyama R., Mardinly A.R., Yamasaki R., Ransohoff R.M., Greenberg M.E., Barres B.A., Stevens B. (2012). Microglia Sculpt Postnatal Neural Circuits in an Activity and Complement-Dependent Manner. Neuron.

[10] Bengtsson M., Ståhlberg A., Rorsman P., Kubista M. (2005). Gene Expression Profiling in Single Cells from the Pancreatic Islets of Langerhans Reveals Lognormal Distribution of MRNA Levels. Genome Res..

[11] Svensson V., Natarajan K.N., Ly L.H., Miragaia R.J., Labalette C., Macaulay I.C., Cvejic A., Teichmann S.A. (2017). Power Analysis of Single-Cell RNA-Sequencing Experiments. Nat. Methods.

[12] Shalek A.K., Satija R., Adiconis X., Gertner R.S., Gaublomme J.T., Raychowdhury R., Schwartz S., Yosef N., Malboeuf C., Lu D. (2013). Single-Cell Transcriptomics Reveals Bimodality in Expression and Splicing in Immune Cells. Nature.

[13] Thrupp N., Frigerio C.S., Wolfs L., Mancuso R., Skene N.G., Fattorelli N., Poovathingal S., Fourne Y., Matthews P.M., Theys T. (2020). Single-Nucleus RNA-Seq Is Not Suitable for Detection of Microglial Activation Genes in Humans. Cell Rep..

[14] Keren-Shaul H., Spinrad A., Weiner A., Matcovitch-Natan O., Dvir-Szternfeld R., Ulland T.K., David E., Baruch K., Lara-Astaiso D., Toth B. (2017). A Unique Microglia Type Associated with Restricting Development of Alzheimer’s Disease. Cell.

[15] Hammond T.R., Dufort C., Dissing-Olesen L., Giera S., Young A., Wysoker A., Walker A.J., Gergits F., Segel M., Nemesh J. (2019). Single-Cell RNA Sequencing of Microglia throughout the Mouse Lifespan and in the Injured Brain Reveals Complex Cell-State Changes. Immunity.

[16] Masuda T., Sankowski R., Staszewski O., Böttcher C., Amann L., Scheiwe C., Nessler S., Kunz P., van Loo G., Coenen V.A. (2019). Spatial and Temporal Heterogeneity of Mouse and Human Microglia at Single-Cell Resolution. Nature.

[17] Jordão M.J.C., Sankowski R., Brendecke S.M., Sagar G.L., Locatelli G., Tai Y.H., Tay T.L., Schramm E., Armbruster S., Hagemeyer N. (2019). Neuroimmunology: Single-Cell Profiling Identifies Myeloid Cell Subsets with Distinct Fates during Neuroinflammation. Science.

[18] Sankowski R., Böttcher C., Masuda T., Geirsdottir L., Sagar, Sindram E., Seredenina T., Muhs A., Scheiwe C., Shah M.J. (2019). Mapping Microglia States in the Human Brain through the Integration of High-Dimensional Techniques. Nat. Neurosci..

[19] Mathys H., Adaikkan C., Gao F., Young J.Z., Manet E., Hemberg M., De Jager P.L., Ransohoff R.M., Regev A., Tsai L.H. (2017). Temporal Tracking of Microglia Activation in Neurodegeneration at Single-Cell Resolution. Cell Rep..

[20] Sousa C., Golebiewska A., Poovathingal S.K., Kaoma T., Pires-Afonso Y., Martina S., Coowar D., Azuaje F., Skupin A., Balling R. (2018). Single-Cell Transcriptomics Reveals Distinct Inflammation-Induced Microglia Signatures. EMBO Rep..

[21] Witcher K.G., Bray C.E., Chunchai T., Zhao F., O’Neil S.M., Gordillo A.J., Campbell W.A., McKim D.B., Liu X., Dziabis J.E. (2021). Traumatic Brain Injury Causes Chronic Cortical Inflammation and Neuronal Dysfunction Mediated by Microglia. J. Neurosci..

[22] Li Y., He X., Kawaguchi R., Zhang Y., Wang Q., Monavarfeshani A., Yang Z., Chen B., Shi Z., Meng H. (2020). Microglia-Organized Scar-Free Spinal Cord Repair in Neonatal Mice. Nature.

[23] Olah M., Menon V., Habib N., Taga M.F., Ma Y., Yung C.J., Cimpean M., Khairallah A., Coronas-Samano G., Sankowski R. (2020). Single Cell RNA Sequencing of Human Microglia Uncovers a Subset Associated with Alzheimer’s Disease. Nat. Commun..

[24] Miedema A., Gerrits E., Brouwer N., Jiang Q., Kracht L., Meijer M., Nutma E., Peferoen-Baert R., Pijnacker A.T.E., Wesseling E.M. (2022). Brain Macrophages Acquire Distinct Transcriptomes in Multiple Sclerosis Lesions and Normal Appearing White Matter. Acta Neuropathol. Commun..

[25] Ziegenhain C., Vieth B., Parekh S., Reinius B., Guillaumet-Adkins A., Smets M., Leonhardt H., Heyn H., Hellmann I., Enard W. (2017). Comparative Analysis of Single-Cell RNA Sequencing Methods. Mol. Cell.

[26] Chen G., Ning B., Shi T. (2019). Single-Cell RNA-Seq Technologies and Related Computational Data Analysis. Front. Genet..

[27] Van Der Maaten L., Hinton G. (2008). Visualizing Data Using T-SNE. J. Mach. Learn. Res..

[28] Mcinnes L., Healy J., Melville J. (2020). UMAP: Uniform Manifold Approximation and Projection for Dimension Reduction. arXiv.

[29] Butovsky O., Jedrychowski M.P., Moore C.S., Cialic R., Lanser A.J., Gabriely G., Koeglsperger T., Dake B., Wu P.M., Doykan C.E. (2014). Identification of a Unique TGF-β Dependent Molecular and Functional Signature in Microglia. Nat. Neurosci..

[30] Torkildsen Ø., Brunborg L.A., Myhr K.M., Bø L. (2008). The Cuprizone Model for Demyelination. Acta Neurol. Scand. Suppl..

[31] Olmstead D.N., Mesnard-Hoaglin N.A., Batka R.J., Haulcomb M.M., Miller W.M., Jones K.J. (2015). Facial Nerve Axotomy in Mice: A Model to Study Motoneuron Response to Injury. J. Vis. Exp..

[32] Constantinescu C.S., Farooqi N., O’Brien K., Gran B. (2011). Experimental Autoimmune Encephalomyelitis (EAE) as a Model for Multiple Sclerosis (MS). Br. J. Pharmacol..

[33] Holtzman D.M., Morris J.C., Goate A.M. (2011). Alzheimer’s Disease: The Challenge of the Second Century. Sci. Transl. Med..

[34] Norden D.M., Godbout J.P. (2013). Review: Microglia of the Aged Brain: Primed to Be Activated and Resistant to Regulation. Neuropathol. Appl. Neurobiol..

[35] Langlois J.A., Rutland-Brown W., Wald M.M. (2006). The Epidemiology and Impact of Traumatic Brain Injury: A Brief Overview. J. Head Trauma Rehabil..

[36] Hyder A.A., Wunderlich C.A., Puvanachandra P., Gururaj G., Kobusingye O.C. (2007). The Impact of Traumatic Brain Injuries: A Global Perspective. NeuroRehabilitation.

[37] Donat C.K., Scott G., Gentleman S.M., Sastre M. (2017). Microglial Activation in Traumatic Brain Injury. Front. Aging Neurosci..

[38] Ximerakis M., Lipnick S.L., Innes B.T., Simmons S.K., Adiconis X., Dionne D., Mayweather B.A., Nguyen L., Niziolek Z., Ozek C. (2019). Single-Cell Transcriptomic Profiling of the Aging Mouse Brain. Nat. Neurosci..

[39] Li Q., Cheng Z., Zhou L., Darmanis S., Neff N.F., Okamoto J., Gulati G., Bennett M.L., Sun L.O., Clarke L.E. (2019). Developmental Heterogeneity of Microglia and Brain Myeloid Cells Revealed by Deep Single-Cell RNA Sequencing. Neuron.

[40] Matcovitch-Natan O., Winter D.R., Giladi A., Aguilar S.V., Spinrad A., Sarrazin S., Ben-Yehuda H., David E., González F.Z., Perrin P. (2016). Microglia Development Follows a Stepwise Program to Regulate Brain Homeostasis. Science.

[41] Calandra T., Roger T. (2003). Macrophage Migration Inhibitory Factor: A Regulator of Innate Immunity. Nat. Rev. Immunol..

[42] Kannan-Thulasiraman P., Seachrist D.D., Mahabeleshwar G.H., Jain M.K., Noy N. (2011). Fatty Acid-Binding Protein 5 and PPARβ/δ Are Critical Mediators of Epidermal Growth Factor Receptor-Induced Carcinoma Cell Growth. J. Biol. Chem..

[43] Liu R.Z., Mita R., Beaulieu M., Gao Z., Godbout R. (2010). Fatty Acid Binding Proteins in Brain Development and Disease. Int. J. Dev. Biol..

[44] Goldmann T., Wieghofer P., Jordão M.J.C., Prutek F., Hagemeyer N., Frenzel K., Amann L., Staszewski O., Kierdorf K., Krueger M. (2016). Origin, Fate and Dynamics of Macrophages at Central Nervous System Interfaces. Nat. Immunol..

[45] Baruch K., Deczkowska A., David E., Castellano J.M., Miller O., Kertser A., Berkutzki T., Barnett-Itzhaki Z., Bezalel D., Wyss-Coray T. (2014). Aging-Induced Type I Interferon Response at the Choroid Plexus Negatively Affects Brain Function. Science.

[46] Grabert K., Michoel T., Karavolos M.H., Clohisey S., Kenneth Baillie J., Stevens M.P., Freeman T.C., Summers K.M., McColl B.W. (2016). Microglial Brain Region−dependent Diversity and Selective Regional Sensitivities to Aging. Nat. Neurosci..

[47] Hagemeyer N., Hanft K.M., Akriditou M.A., Unger N., Park E.S., Stanley E.R., Staszewski O., Dimou L., Prinz M. (2017). Microglia Contribute to Normal Myelinogenesis and to Oligodendrocyte Progenitor Maintenance during Adulthood. Acta Neuropathol..

[48] Leyns C.E.G., Holtzman D.M. (2017). Glial Contributions to Neurodegeneration in Tauopathies. Mol. Neurodegener..

[49] Hong S., Beja-Glasser V.F., Nfonoyim B.M., Frouin A., Li S., Ramakrishnan S., Merry K.M., Shi Q., Rosenthal A., Barres B.A. (2016). Complement and Microglia Mediate Early Synapse Loss in Alzheimer Mouse Models. Science.

[50] Ritzel R.M., Patel A.R., Pan S., Crapser J., Hammond M., Jellison E., McCullough L.D. (2015). Age- and Location-Related Changes in Microglial Function. Neurobiol. Aging.

[51] Denisenko E., Guo B.B., Jones M., Hou R., De Kock L., Lassmann T., Poppe D., Poppe D., Clément O., Simmons R.K. (2020). Systematic Assessment of Tissue Dissociation and Storage Biases in Single-Cell and Single-Nucleus RNA-Seq Workflows. Genome Biol..

[52] Marsh S.E., Walker A.J., Kamath T., Dissing-Olesen L., Hammond T.R., de Soysa T.Y., Young A.M.H., Murphy S., Abdulraouf A., Nadaf N. (2022). Dissection of Artifactual and Confounding Glial Signatures by Single-Cell Sequencing of Mouse and Human Brain. Nat. Neurosci..

[53] Geirsdottir L., David E., Keren-Shaul H., Weiner A., Bohlen S.C., Neuber J., Balic A., Giladi A., Sheban F., Dutertre C.A. (2019). Cross-Species Single-Cell Analysis Reveals Divergence of the Primate Microglia Program. Cell.

[54] Esaulova E., Cantoni C., Shchukina I., Zaitsev K., Bucelli R.C., Wu G.F., Artyomov M.N., Cross A.H., Edelson B.T. (2020). Single-Cell RNA-Seq Analysis of Human CSF Microglia and Myeloid Cells in Neuroinflammation. Neurol. Neuroimmunol. Neuroinflamm..

